# Non-response bias in physical activity trend estimates

**DOI:** 10.1186/1471-2458-9-425

**Published:** 2009-11-22

**Authors:** Cora L Craig, Christine Cameron, Joe Griffiths, Adrian Bauman, Catrine Tudor-Locke, Ross E Andersen

**Affiliations:** 1 Canadian Fitness and Lifestyle Research Institute, Ottawa, Canada; 2 School of Public Health, University of Sydney, Australia; 3 Pennington Biomedical Research Center, Baton Rouge, USA; 4 Department of Kinesiology and Physical Education, McGill University, Montreal, Canada

## Abstract

**Background:**

Increases in reported leisure time physical activity (PA) and obesity have been observed in several countries. One hypothesis for these apparently contradictory trends is differential bias in estimates over time. The purpose of this short report is to examine the potential impact of changes in response rates over time on the prevalence of adequate PA in Canadian adults.

**Methods:**

Participants were recruited in representative national telephone surveys of PA from 1995-2007. Differences in PA prevalence estimates between participants and those hard to reach were assessed using Student's t tests adjusted for multiple comparisons.

**Results:**

The number of telephone calls required to reach and speak with someone in the household increased over time, as did the percentage of selected participants who initially refused during the first interview attempt. A higher prevalence of adequate PA was observed with 5-9 attempts to reach anyone in the household in 1999-2002, but this was not significant after adjustment for multiple comparisons.

**Conclusion:**

No significant impact on PA trend estimates was observed due to differential non response rates. It is important for health policy makers to understand potential biases and how these may affect secular trends in all aspects of the energy balance equation.

## Background

The Global Strategy for Diet and Physical Activity [1] emerged in response to the recognition of the growing global burden posed by non communicable disease (NCD). Many national health surveys monitor NCD risk factors using self-reported data to assess trends in these risk factors over time. [2-5].

The physical activity levels of Canadian have been tracked since 1981 [6]. These data have been essential in setting national physical activity goals, identifying which groups are less active than others and determining whether differences in physical activity levels between groups have been increasing, decreasing or remaining stable over time. In Canada, population levels of leisure-time physical activity (PA) based on self-reports have increased over a twenty-year period. [6] However, during this same period the prevalence of overweight and obesity has also increased at alarming rates. This co-occurrence of increases in reported leisure time PA and obesity has also been observed in Finland [7,8], Spain [9], Australia [10,11] and elsewhere. These apparently contradictory trends may be explained by changes in dietary patterns, the limitation of physical activity measures based on only leisure-time rather than total activity across all domains, or of other changing patterns. Other explanations include differences in response bias, such as changes in social desirability bias regarding active lifestyles, or decreasing response rates (for example due to the increase in telemarketing) leading to differential non-response bias in estimates over time [12]. Differentials in the magnitude of bias over time may lead to erroneous conclusions regarding the changes in the monitored prevalence estimates and relationships with other variables of interest.

The purpose of this short report is to examine potential non-response biases (e.g., in terms of number of attempted contacts) in self-reported data introduced during the recruitment to random digit dial surveys and how these may influence trend estimates, using the example of PA.

## Methods

Study participants 18 years and older were recruited to the Physical Activity Monitor (PAM) a nationally representative Canadian survey of adults launched in 1995, repeated in 1997 and run continuously since 1998 employing a monthly sample. The sample is selected in two steps. Households are selected using random digit dialing and then the individual with the nearest birth date to the interview date is selected. During the recruitment process, the following information is recorded 1) number of attempts made to call the household, 2) number of times someone answered in trying to reach the selected individual, 3) the number of contacts with the selected individual and completion status (initial agreement, refusal converted to participation). Verbal informed consent was obtained according to the procedures of the Ethics Committee, York University.

An adapted version of the Minnesota Leisure Time Physical Activity questions was administered from 1995-2002 and then again in 2007. The PA score was calculated by summing the product of the number of times each type of PA was performed in the previous 12 months, the duration in minutes and the intensity of the activity expressed in METs [13]. 'Adequate' PA was defined as 5294 kilojoules (1260 MET-minutes) per week and equivalent to at least 60 minutes of moderate-intensity activity daily [14]. Differences in the prevalence of adequate PA by attempts, answers, contacts, and completion status were tested across and within survey periods using Student's t tests (SPSS Complex Samples, version15) and adjusting significance levels for multiple comparisons by Holm's procedure.

## Results

In total, 26,496 individuals were involved in the study between 1995 and 2007. Overall, the number of calls required to reach someone in the selected households increased over the period (Table 1). In 1995-1998, 10 or more such calls were required to reach 13.2% of respondents and this percentage increased to 23.3% by 2007. Similarly, the number of times that the calls were answered by someone in the household (to select individual respondents and then contact them) increased, with 3 or more calls being required in 41.6% of households in 1995-1998 compared to 59.8% in 2007. After selecting the respondent, at least three contacts with that respondent were made to complete the interview (e.g., due to inconvenient time of call) in 15.3% of households in 2007, which was higher than the percentages required in 1999-2002. Agreeing to participate in the survey during the first attempt to interview the respondent decreased over the twelve year span, with initial refusals roughly twice as high in 2007 compared to the early and middle years.

**Table 1:** Distribution of adults responding to Canadian Physical Activity Monitor by stages in participant recruitment, 1995-2007

	% (95% CI)	1995-1998 % (CI*)	1999-2002 % (CI*)	2007 % (CI*)
Number of calls made to reach the someone in the household				
1 to 2	37.6 (36.4-38.7)	38.5 (36.4-40.6)	38.2 (36.6-39.8)	22.0 (17.6-27.3)
3 to 4	23.8 (23.1-24.6)	24.2 (22.9-25.5)	23.3 (22.3-24.3)	26.7 (23.4-30.3)
5 to 9	24.7 (24.0-25.5)	24.1 (22.9-25.4)	24.9 (23.9-25.9)	27.9 (24.8-31.4)
10 or more	13.9 (13.3-14.5)	13.2 (11.9-14.7)	13.6 (12.5-14.8)	23.3 (19.0 28.2)

Number of calls answered by someone in the household				
1	36.2 (35.4-37.0)	35.6 (33.7-37.6)	39.1 (37.6-40.7)	6.9 (4.8-9.9)
2	23.6 (22.8-24.3)	22.8 (21.2-24.5)	23.3 (22.1-24.5)	33.3 (28.7-38.3)
3 or more	40.2 (39.4-41.1)	41.6 (39.7-43.5)	37.6 (36.1-39.2)	59.8 (54.6-64.9)

Number of contacts with the selected respondent				
1	73.4 (72.6-74.2)	73.4 (71.6-75.2)	78.0 (76.6-79.3)	20.2 (16.3-24.8)
2	15.9 (15.2-16.5)	14.8 (13.5-16.3)	12.5 (11.5-13.5)	64.5 (59.3-69.3)
3 or more	10.7 (10.2-11.3)	11.8 (10.6-13.1)	9.5 (8.7-10.4)	15.3 (12.1-19.3)

Final completion status				
Agreed to participate during 1st contact	88.5 (87.9-89.1)	88.8 (87.6-89.8)	89.5 (88.6-90.4)	74.5 (70.4-78.3)
Initial refusal; converted to completion by senior interviewer	11.5 (10.9-12.1)	11.2 (10.2-12.4)	10.5 (9.6-11.4)	25.5 (21.7-29.6)

* 95% Confidence Intervals with Holm adjustments for multiple comparisons

Prevalence of 'adequate PA' was unrelated to the number of attempts made to contact someone in the household in 1995-1998 and 2007. In 1999-2002 only, a higher prevalence of adequate PA was observed with 5-9 attempts than in fewer attempts (and compared to 5-9 attempts in 1995-98), but this was not significant after adjusting for multiple comparisons (Table 2). At all time periods, adequate PA was unrelated to the number of times someone in the household answered the telephone, the number of times the selected individual was contacted, and whether that individual initially agreed or was converted from a refusal to a participant.

**Table 2:** Adequate physical activity (PA) among Canadian adults by stages in participant recruitment, 1995-2007

	n	1995-1998 % (CI*)	1999-2002 % (CI*)	2007 % (CI*)
Number of calls made to reach the someone in the household				
1 to 2	10,394	35.5 (33.4-37.7)	38.3 (36.6-40.1)	41.6 (33.2-50.6)
3 to 4	6182	34.4 (31.8-37.2)	38.2 (36.0-40.6)	32.9 (26.1-40.4)
5 to 9	6493	35.9 (32.1-39.9)	42.9 (39.6-46.3)	43.4 (36.7-50.2)
10 or more	3427	35.4 (31.9-39.0)	40.1 (37.0-43.2)	39.1 (31.7-47.0)

Number of calls answered by someone in the household				
1	10 372	36.9 (34.8-39.1)	40.6 (38.9-42.3)	29.7 (19.3-42.8)
2	6114	33.9 (31.1-36.7)	38.0 (35.8-40.3)	44.0 (37.3-51.1)
3 or more	10 010	34.8 (32.8-36.9)	39.8 (37.9-41.7)	37.6 (33.0-42.4)

Number of contacts with the selected respondent				
1	19 570	36.0 (34.5-37.6)	40.5 (39.2-41.7)	40.7 (32.9-49.0)
2	4186	35.6 (32.3-39.1)	37.0 (34.0-40.2)	37.9 (33.3-42.8)
3 or more	2740	30.8 (27.2-34.5)	36.4 (32.9-40.0)	40.8 (31.6-50.7)

Final completion status				
Agreed to participate during 1st contact	23813	35.8 (34.4-37.2)	40.1 (39.0-41.3)	39.2 (34.9-43.7)
Initial refusal; converted to completion by senior interviewer	2683	31.7 (28.0-35.7)	35.7 (32.4-39.2)	39.6 (32.2-46.5)

* 95% Confidence Intervals with Holm adjustments for multiple comparisons

## Discussion

This study found no evidence of a differential bias in levels of adequate PA according to the various stages at which non-response could occur during recruitment. Therefore, there is no support for the hypothesis that increase in physical activity over time were related to changes in the underlying representivity of the samples. As a result, differences in non-response rates do not explain the apparent contradictory trends of increases in both sufficient leisure time physical activity and obesity over time. More investigation is required to understand changes in the multiple factors influencing the energy balance equation. In particular, a better understanding is required of changes in sedentary behaviours, total energy expenditure through physical activity, dietary patterns, and total energy intake, and how these changes relate to the rising rates of overweight and obesity.

One limitation of this study is that comparisons were made between individuals who were easier and harder to recruit, but was unable to make comparisons with those not recruited at all due to a lack of information external to the surveys. External data has been used to quantify non-response bias in other studies with equivocal results. Using proxy reports from more cooperative or readily accessible family members for PA and other key variables, Vink [15] determined that although the non responding family members generally had less favorable estimates than those responding, the impact on estimates was small. In the Netherlands, the prevalence of healthy lifestyle behaviours was over estimated, but there was no bias in associations [16]. In contrast, Macera et al [17] found limited impact on estimates of key variables comparing an initial face-to-face contact (as opposed to telephone contact herein) to subsequent follow-up. This was also the case in a different Canadian study using the same PA measure as in the current study [18]. However, trend estimates may also be influenced by response bias including changes in question order, inclusion of sensitive subject areas in surveys, and changes in social desirability (for example, higher over-reporting of PA as low levels of participation become more widely recognized as a health issue). Accounting for bias is important to those involved in health policy to ensure that any observed changes are indeed due to secular trends and not an artifact of temporal differences in bias.

## Conclusion

Although it was not possible to quantify differential impacts of response bias over time, the data do not demonstrate a significant effect on trend estimates due to differential response rates over the twelve year span.

## Competing interests

The authors declare that they have no competing interests.

## Authors' contributions

CLC conceived this study, conceived the original study on which it is based, and wrote the manuscript. CC acquired the data, and contributed to interpretation of results and to the writing of the manuscript. JG assisted in the analysis and critically reviewed earlier drafts of the manuscript. AB contributed to the conceptualization of this study and interpretation of findings, and contributed to the writing. CTL and REA assisted conceptualize this study and provided substantial revision of earlier drafts of the manuscript. All authors have read and approved the final manuscript.

## Pre-publication history

The pre-publication history for this paper can be accessed here:

http://www.biomedcentral.com/1471-2458/9/425/prepub
